# Leucine-rich alpha-2-glycoprotein 1 (LRG1) decrement during biologics therapy and its correlation with disease features and treatment outcomes in rheumatoid arthritis patients

**DOI:** 10.1007/s10067-025-07545-2

**Published:** 2025-07-15

**Authors:** Liang Zou, Qiuyu Fan, Ya Liu, Hao He, Chao Jia

**Affiliations:** Department of Rheumatology and Immunology, Wuhan No.1 Hospital, No.215 Zhongshan Avenue, Wuhan, 430022 Hubei China

**Keywords:** Disease activity, Health controls, Response to biologics, Rheumatoid arthritis, Serum LRG1

## Abstract

**Objectives:**

Leucine-rich alpha-2-glycoprotein 1 (LRG1) was previously reported to regulate inflammation and arthritis progression. This study aimed to investigate the correlation of serum LRG1 level with disease features and response to biologics in rheumatoid arthritis (RA) patients.

**Methods:**

Seventy-eight RA patients who underwent biologics treatment were analyzed. Serum LRG1 level was detected by enzyme-linked immunosorbent assay at baseline (before biologics were initiated) and at weeks 6 and 12. Treatment response, low disease activity (LDA), and remission were analyzed on the basis of disease activity score in 28 joints. Moreover, serum LRG1 level in another 20 health controls was also analyzed.

**Results:**

LRG1 was greater in RA patients than in health controls (46.3 versus 28.6 µg/mL, *P* < 0.001), with an area under the curve of 0.795 for differentiating them according to the receiver operator characteristic curve. By correlation analysis, LRG1 was correlated with a greater body mass index (*P* = 0.007) and C-reactive protein level (*P* = 0.013) in RA patients and tended to be associated with swollen joint count but was not statistically significant (*P* = 0.052). Furthermore, LRG1 decreased from baseline to week 12 after biologics treatment in RA patients (*P* < 0.001). However, baseline LRG1 was not correlated with treatment response (*P* = 0.987), LDA (*P* = 0.405), or remission (*P* = 0.763) in RA patients. A decrease in LRG1 at week 12 (*P* = 0.028) was related to response achievement, and a decrease in LRG1 at week 6 (*P* = 0.047) and week 12 (*P* = 0.019) was related to LDA achievement.

**Conclusion:**

LRG1 may aid in RA disease supervision, but further validation is needed.

**Table:** 

**Key Points** • *LRG1 level can distinguish RA patients from health controls with a high AUC at 0.795.* • *LRG1 level is correlated with higher BMI and CRP level, and tends to be related to elevated SJC in RA patients.* • *LRG1 level after treatment is correlated with clinical response and LDA to biologics in RA patients, while its level before treatment fails to do so.* • *Collectively, LRG1 level shows a potential to be a biomarker for RA disease supervision.*

## Introduction

Rheumatoid arthritis (RA) is a chronic autoimmune disease that affects mainly females, with an estimated global prevalence of 0.46% [1, 2]. Profiting from the progress of the biopharmaceutical industry, several novel kinds of drugs have been developed in recent years, such as biologics or biosimilars (etanercept, adalimumab, infliximab, golimumab, tocilizumab, etc.) and targeted synthetic disease-modifying anti-rheumatic drugs (DMARDs) (tofacitinib, baritinib, etc.), which greatly improve the outcomes of RA patients [3–5]. However, a nonnegligible number of RA patients insufficiently respond to these therapies or relapse/flare early after achieving an initial response [6–8].

To help resolve this issue, the identification of markers that can predict the treatment response to identify risky patients in advance is a possible solution that can be used to personalize treatment drugs and strategies to improve RA patients’ outcomes [9, 10]. For example, the plasma resistin level is positively correlated with disease activity and can predict 5-year radiological progression in RA patients treated with methotrexate, sulfasalazine, hydroxychloroquine, or prednisolone with or without infliximab [11]; the serum JNK pathway-associated phosphatase (JKAP) level is inversely associated with multiple inflammatory and disease-activity-related indexes, and its increase over 24 weeks is related to the response to etanercept in RA patients [12]. These findings imply the importance of biomarkers for RA management.

Leucine-rich alpha-2-glycoprotein 1 (LRG1) is a glycoprotein with a carbohydrate content of 23% and 5 estimated glycosylation sites that widely modulates immune, proinflammatory, metabolic, fibrotic, oncogenic, and vasculopathic biological processes [13]. Specific to immune and inflammatory regulation, LRG1 is reported to regulate TGF-β-mediated hematopoietic progenitor functions, neutrophil activation and extravasation, M1 macrophage polarization, and Th17 cell proliferation [14–18]. Furthermore, LRG1 is elevated quickly in the blood following inflammatory stimulation [19, 20]. LRG1 is also dysregulated in several autoimmune diseases, such as psoriasis, lupus nephritis, vasculitis, and RA [21–24]. Moreover, a very recent study reported that LRG1 was correlated with C-reaction protein (CRP), erythrocyte sedimentation rate (ESR), and traditional biomarkers in RA patients receiving IL-6 inhibitor, which remained elevated despite suppression of CRP and ESR, indicating it might serve as an alternative disease activity biomarker for RA [24]. However, its longitude variation and correlation with the response to biologics is still unclear.

Therefore, the present study aimed to detect the serum LRG1 level variation during treatment, and explore its correlation with disease risk, activity, and response to biologics in RA patients.

## Methods

### Patients

This study consecutively included 78 RA patients underwent biologics treatment. The inclusion criteria were as follows: (i) diagnosed as RA according to ACR/EULAR 2010 criteria, (ii) at moderate-severe active disease status defined as 28-joint disease activity score using ESR (DAS28_ESR_) above 3.2, (iii) about to initiate biologics treatment, (iv) considered to be able to be followed up regularly for at least 3 months, and (v) signed the informed consent. The exclusion criteria were as follows: (i) complicated with malignancies or other inflammatory diseases, (ii) complicated with active infection or hepatitis B, (iii) was taking the biologics or targeted synthetic DMARDs at the time of enrollment, and (iv) pregnancies or during lactation period. In addition, another 20 health controls with age and gender matched to RA patients were included, which were defined as no abnormities (including comorbidities) by physical examination. The Ethics Review Borad of Wuhan No.1 Hospital approved this study.

### Data collection, treatment, and outcomes

The baseline data of RA patients, which included demographics, treatment history, antibody positivity, and disease activity indexes, were collected. RA patients underwent biologics treatment, including adalimumab, etanercept, and infliximab. For adalimumab, 40 mg of drug was given every 2 weeks; for etanercept, 25 mg of drug was given twice every week, or 50 mg of drug was given once every week; for infliximab, 3 mg/kg drug was given at 0, 2, and 6 weeks and then every 8 weeks. DAS28_ESR_ was assessed at week 0, week 6 (±1 week), and week 12 (±2 weeks) after the initiation of biologics treatment as outcomes. Patients who did not have follow-up DAS28_ESR_ data were excluded from the analysis. In brief, seven patients lost to follow-up and had been already excluded from the analysis of this study.

### Definitions

Response was defined as a decline in DAS28_ESR_ ≥ 2.0 from baseline. Low disease activity (LDA) was defined as a DAS28_ESR_ < 3.2 and ≥ 2.6. Remission was defined as a DAS28_ESR_ < 2.6. The treatment response, LDA, and remission were assessed at week 6 and week 12 according to the DAS28_ESR_.

### LRG1 measurement

Serum was acquired at week 0, week 6, and week 12 from RA patients and at inclusion in health controls. LRG1 was then detected by the enzyme-linked immunosorbent assay (ELISA) kits (Elabscience, China).

### Statistical analysis

SPSS 26.0 (IBM, USA) was used for statistical analysis. The data are presented as numbers (%), means ± standard deviations, or medians (interquartile ranges). Comparisons were analyzed via the Wilcoxon rank sum test or the Friedman test. Correlations were analyzed via the Spearman test. The ability of LRG1 to distinguish RA patients from health controls was analyzed via receiver operator characteristic (ROC) curves. *P* < 0.05 was considered statistically significant.

## Results

### Characteristics of RA patients

The analyzed RA patients consisted of 82.1% females and 17.9% males, with an age of 53.5 ± 11.6 years (Table 1). The disease duration of RA was 5.0 (2.4–11.3) years, all patients had treatment history of conventional DMARDs, 10.3% patients had treatment history of targeted synthetic DMARDs, and 21.8% of patients had undergone biologics treatment. The baseline DAS28_ESR_ was 5.6 ± 0.8 and the baseline clinical disease activity index (CDAI) was 25.3 ± 9.3.

**Table 1:** RA patients’ characteristics

Items	RA patients (*N* = 78)
Age (years)	53.5 ± 11.6
Sex	
Females	64 (82.1%)
Males	14 (17.9%)
BMI (kg/m^2^)	22.9 ± 3.1
Treatment history	
Conventional DMARDs	78 (100.0%)
Targeted synthetic DMARDs	8 (10.3%)
Biologics	17 (21.8%)
RF status	
Positive	68 (87.2%)
Negative	10 (12.8%)
ACPA status	
Positive	71 (91.0%)
Negative	7 (9.0%)
Disease duration (years)	5.0 (2.4–11.3)
Baseline disease activity assessment	
TJC (counts)	8.3 ± 4.4
SJC (counts)	5.8 ± 3.2
ESR (mm/h)	53.5 (36.7–75.7)
CRP (mg/L)	32.7 (20.0–59.9)
DAS28_ESR_ (points)	5.6 ± 0.8
PGA (points)	5.7 ± 1.5
PhGA (points)	5.5 ± 1.6
CDAI (points)	25.3 ± 9.3

*RA* rheumatoid arthritis, *BMI* body mass index, *DMARDs* disease-modifying anti-rheumatic drugs, *RF* rheumatoid factor, *ACPA* anti-citrullinated protein autoantibody, *TJC* tender joint count, *SJC* swollen joint count, *ESR* erythrocyte sedimentation rate, *CRP* C-reactive protein, *DAS28*_*ESR*_ 28-joint disease activity score using erythrocyte sedimentation rate, *PGA* patient’s global assessment, *PhGA* physician’s global assessment, *CDAI* clinical disease activity index

### LRG1 level in RA patients

**Fig. 1:**
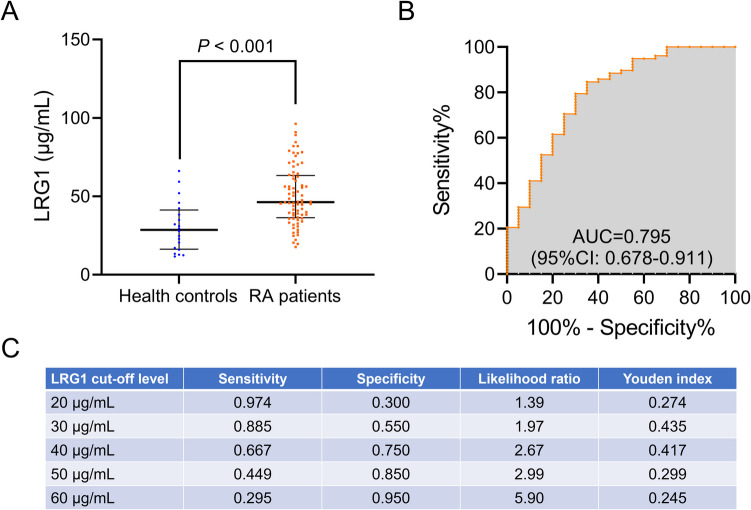
Dysregulated LRG1 level. Comparison of LRG1 level between RA patients and health controls (**A**). ROC curve analysis of LRG1 level for distinguishing RA patients from health controls (**B**). Diagnostic indexes of LRG1 level by different cutoffs for RA (**C**)

LRG1 level was 46.3 (36.3–63.2) µg/mL in RA patients, which was much higher than that in health controls (28.6 (16.2–41.3) µg/mL, *P* < 0.001, Fig. 1A). The ROC curve revealed that LRG1 had an acceptable ability to distinguish RA patients from health controls (area under the curve (AUC) = 0.795, 95%CI: 0.678–0.911, Fig. 1B). By cutting off at 20, 30, 40, 50, and 60 µg/mL of LRG1 level, LRG1 threshold of 30 µg/mL showed the highest Youden index of 0.435 (Fig. 1C).

### Correlation between LRG1 level and characteristics in RA patients

LRG1 level was positively correlated with body mass index (BMI, *r* = 0.305, *P* = 0.007) and CRP (*r* = 0.279, *P* = 0.013), and tended to be associated with swollen joint count (SJC) but not statistically significant (*r* = 0.221, *P* = 0.052) (Table 2). However, LRG1 was not correlated with other characteristics in RA patients.

**Table 2:** Correlation between LRG1 level and RA patients’ characteristics

Items	*r* correlation coefficient	*P* value
Age	0.124	0.279
Sex-females	0.108	0.345
BMI	0.305	0.007
Treatment history		
Conventional DMARDs	-	-
Targeted synthetic DMARDs	0.058	0.613
Biologics	0.020	0.862
RF status-positive	0.136	0.234
ACPA status-positive	0.178	0.118
Disease duration	0.152	0.183
Baseline disease activity assessment		
TJC	0.153	0.181
SJC	0.221	0.052
ESR	0.093	0.418
CRP	0.279	0.013
DAS28_ESR_	0.178	0.119
PGA	0.133	0.245
PhGA	0.125	0.275
CDAI	0.200	0.078

*LRG1* leucine rich alpha-2-glycoprotein 1, *RA* rheumatoid arthritis, *BMI* body mass index, *DMARDs* disease-modifying anti-rheumatic drug, *RF* rheumatoid factor, *ACPA* anti-citrullinated protein autoantibody, *TJC* tender joint count, *SJC* swollen joint count, *ESR* erythrocyte sedimentation rate, *CRP* C-reactive protein, *DAS28*_*ESR*_ 28-joint disease activity score using erythrocyte sedimentation rate, *PGA* patient’s global assessment, *PhGA* physician’s global assessment, *CDAI* clinical disease activity index

### Treatment information and outcomes in RA patients

All RA patients started biologics treatment, among them 43.6% of patients received adalimumab treatment, 42.3% of patients received etanercept treatment, and 14.1% of patients received infliximab treatment (Table 3). In addition, 96.2% of patients received the combination of conventional DMARDs.

**Table 3:** Current treatment information in RA patients

Items	RA patients (*N* = 78)
Initiation of biologics	78 (100.0%)
Type of biologics	
Adalimumab	34 (43.6%)
Etanercept	33 (42.3%)
Infliximab	11 (14.1%)
Combined conventional DMARDs	75 (96.2%)

*RA* rheumatoid arthritis, *DMARDs* disease-modifying anti-rheumatic drug

**Fig. 2:**
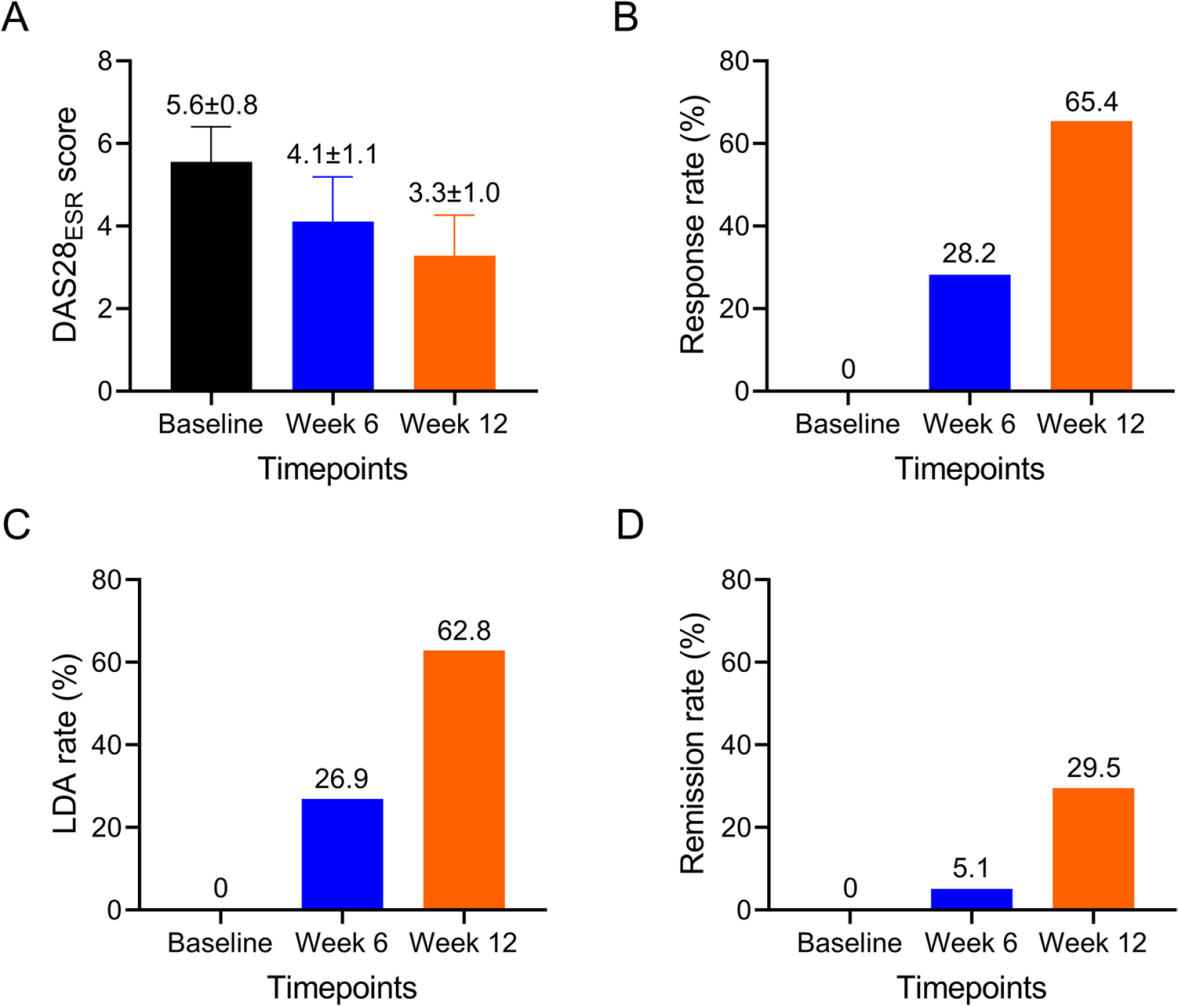
Treatment outcomes at different timepoints. DAS28_ESR_ score at baseline, week 6, and week 12 (**A**). Response rate at baseline, week 6, and week 12 (**B**). LDA rate at baseline, week 6, and week 12 (**C**). Remission rate at baseline, week 6, and week 12 (**D**)

The DAS28_ESR_ gradually decreased from 5.6 ± 0.8 at baseline, and to 4.1 ± 1.1 at week 6, to 3.3 ± 1.0 at week 12 in RA patients (Fig. 2A). A total of 28.2% and 65.4% of RA patients realized treatment response at week 6 and week 12, respectively (Fig. 2B); 26.9% and 62.8% RA patients realized LDA at week 6 and week 12, respectively (Fig. 2C); 5.1% and 29.5% RA patients realized remission at week 6 and week 12, respectively (Fig. 2D).

### Correlation between LRG1 decrement during biologics treatment and patients’ outcomes

**Fig. 3:**
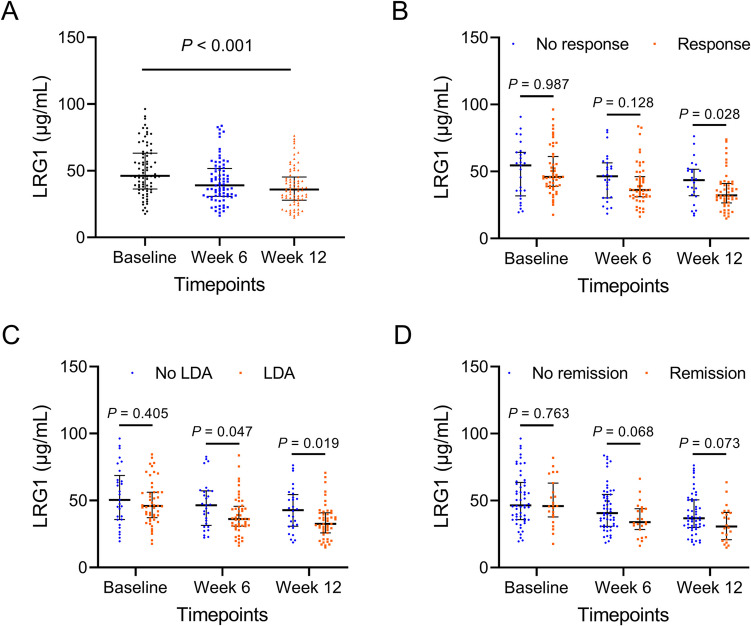
LRG1 decrement related to treatment outcome to some extent. LRG1 level at baseline, week 6, and week 12 (**A**). Comparison of LRG1 level at baseline, week 6, and week 12, between response RA patients and no response RA patients (**B**), between LDA RA patients and no LDA RA patients (**C**), between remission RA patients and no remission RA patients (**D**)

LRG1 level was gradually decreased from baseline to week 12 in RA patients (*P* < 0.001, Fig. 3A). After comparison, LRG1 level at baseline (*P* = 0.987) and week 6 (*P* = 0.128) was not different, while its level at week 12 (*P* = 0.028) was lower, in response patients versus no response patients (Fig. 3B). LRG1 level at baseline (*P* = 0.405) was not varied, but its level at week 6 (*P* = 0.047) and week 12 (*P* = 0.019) was reduced, in LDA patients versus no LDA patients (Fig. 3C). In addition, LRG1 level at any timepoints was not different between remission patients and no remission patients; but it should be stated that LRG1 level at week 6 (*P* = 0.068) and week 12 (*P* = 0.073) showed a tendency to be lower in remission patients versus no remission patients, even though not statistically (Fig. 3D).

## Discussion

LRG1 has been shown to be aberrantly expressed in several autoimmune diseases [21, 25, 26]. For example, serum LRG1 level was elevated in psoriasis patients and psoriatic arthritis patients compared to health controls [21]; serum LRG1 level was also increased in ulcerative colitis patients compared with health controls [25]. Regarding RA, a study revealed that LRG1 level was much greater in RA patients than that in health controls [26]. This study revealed that serum LRG1 level was elevated in RA patients compared to health controls, which was in line with the findings of a previous study [26]. In addition, serum LRG1 was able to well distinguish RA patients from health controls (AUC = 0.795); moreover, with a cutoff level of 30 µg/mL, LRG1 showed diagnostic utility for RA, with a sensitivity of 0.885, a specificity of 0.550, and a Youden index of 0.435. These findings indicated that LRG1 had the potential to be a diagnostic biomarker for RA.

LRG1 has also been shown to be correlated with disease severity indexes in autoimmune diseases [25–27]. For example, serum LRG1 is positively correlated with both clinical activity and endoscopic activity in ulcerative colitis patients [25]; it is also positively associated with CRP level, ferritin level, and disease activity in systemic juvenile idiopathic arthritis patients [27]. In terms of RA, a previous study reported that serum LRG1 was correlated with higher DAS28, CRP, and ESR levels [26]. In this study, serum LRG1 was positively correlated with BMI and CRP, and tended to be associated with SJC but not statistically. These findings were partially in accordance with those of a previous study [26]. The correlation between LRG1 and BMI could be explained by the relationship of LRG1 with thyroid hormones, insulin, angiogenesis, and obesity-related markers [28–32]. The correlation between LRG1 and CRP could be explained by the relationship between LRG1 and inflammation [33]. Furthermore, a latest study reported that LRG1 was associated with CRP, ESR, and traditional biomarkers, but remained high despite suppression of CRP and ESR in RA patients receiving IL-6 inhibitor [24]. Compared to the abovementioned previous study, this study found that LRG1 level was much higher in RA patients than that in health controls, and it had potential as a biomarker aiding in RA diagnosis. In addition, this study also explored the longitude variation of LRG1 during treatment and its correlation with the response to biologics, which provided new evidence of LRG1 as a potential biomarker for RA.

Regarding the correlation between LRG1 level and treatment outcomes in patients with autoimmune diseases, only one previous study reported this topic, which revealed that LRG1 gene expression was dysregulated in canakinumab responders compared with canakinumab non-responders among systemic juvenile idiopathic arthritis patients [34]. This study revealed that LRG1 level gradually decreased from baseline to week 12 in RA patients who underwent biologics treatment, which could be explained by the anti-inflammatory effect of biologics on the level of LRG1 (an inflammatory marker) [35–37]. In addition, and importantly, a decrease in LRG1 was related to response and LDA to biologics treatment in RA patients and tended to be related to remission (not statistically significant), indicating the potential of LRG1 as a prognostic marker for biologics in RA patients. Moreover, LRG1 decrement reflected on-treatment response more than pre-treatment prediction. However, more data and evidences are needed for validation. It could be mentioned that DAS28_CRP_ may underestimate disease activity and overestimate EULAR response criteria compared to DAS28_ESR_ in RA patient [38], and DAS28_ESR_ is commonly applied in our clinical practice and in previous studies [39, 40], DAS28_ESR_ was applied for assessment in this study.

The limitations of this study include the following: (i) The limited sample size restricted the generalizability of the study findings. (ii) RA patients receiving biologics were analyzed; therefore, the clinical value of LRG1 in RA patients receiving other treatment regimens is not clear. (iii) A 12-week follow-up period made radiographic progression evaluation unnecessary; therefore, the relationship of LRG1 level with radiographic progression risk in RA patients could be evaluated in future studies.

In conclusion, LRG1 may aid in RA disease supervision and biologics treatment response prediction, but further validation is needed.

## Data Availability

The datasets generated and analyzed during the current study are available from the corresponding author on reasonable request.
